# Ischemic postconditioning prevents renal ischemia reperfusion injury through the induction of heat shock proteins in rats

**DOI:** 10.3892/mmr.2014.2641

**Published:** 2014-10-14

**Authors:** QIONGMEI GUO, XUEFANG DU, YANLI ZHAO, DONG ZHANG, LIHUI YUE, ZHENXIAN WANG

**Affiliations:** 1 Department of Anesthesiology, Hebei General Hospital, Shijiazhuang, Hebei 050051, P.R. China; 2 Department of Anesthesiology, The First Hospital of Hebei Medical University, Shijiazhuang, Hebei 050053, P.R. China; 3 Department of Anesthesiology, Xingtai Eye Hospital, Xingtai, Hebei 054001, P.R. China; 4 Department of Urinary Surgery, Hebei General Hospital, Shijiazhuang, Hebei 050051, P.R. China

**Keywords:** heat shock protein 70, heat shock protein 27, heme oxygenase 1, postconditioning, reperfusion injury, renal

## Abstract

Ischemic postconditioning (IPo) attenuates ischemia-reperfusion injuries (IRI) in various organs, of both animals and humans. This study tested the hypothesis that IPo attenuates renal IRI through the upregulation of heat shock protein (HSP)70, HSP27 and heme oxygenase-1 (HO-1, also known as HSP 32) expression. Adult Sprague Dawley rats were subjected to bilateral renal ischemia for 45 min followed by reperfusion for up to 48 h. One group of rats received IPo prior to restoring full perfusion. Another group was administered 100 mg/kg HSP inhibitor quercetin, injected intraperitoneally 1 h prior to ischemia. Control rats received sham operations. Renal IR resulted in severe morphological and pathological changes, with increased serum creatinine and blood urea nitrogen concentrations. IR resulted in increased inflammation by inducing plasma tumor necrosis factor-α and renal nuclear factor kappa-light-chain-enhancer of activated B cells expression. IR also increased lipid peroxidation, as indicated by elevated malondialdehyde content, reduced superoxide dismutase activity and increased renal apoptosis. Renal HSP70, HSP27 and HO-1 mRNA and protein levels were increased by IR and further elevated by IPo. IPo attenuated these changes observed in pathology, lipid peroxidation, apoptosis and inflammation. Quercetin treatment abolished all the protective effects of IPo. In conclusion, this study showed that IPo can attenuate lipid peroxidation, apoptosis and inflammation as well as renal IRI by upregulating the expression of HSP70, HSP27 and HO-1.

## Introduction

Due to their high levels of reperfusion, the kidneys are prone to ischemia reperfusion injury (IRI), and this condition may cause or aggravate renal dysfunction. Renal ischemia may be caused by arterial occlusion, shock and organ transplantation, and leads to renal cell death, renal failure, delayed graft function and graft rejection (1). These events contribute substantially to renal-associated morbidity and mortality, with a 30–50% death rate, following acute renal failure (ARF) (2). In addition, ~10% of renal allografts fail during the first year after transplantation, and the risk increases by 3–5% each year (3). Since the first report by Zhao *et al* (4), several recent studies have shown that brief ischemia during the onset of reperfusion, ischemic postconditioning (IPo) is protective in various organs (5–7). IPo has therefore become a clinical intervention to significantly reduce IRI (8–10). In a previous study by our group (11), it was demonstrated that IPo significantly reduced renal IRI by attenuating renal lipid peroxidation and cell apoptosis (11). Miklós *et al* (12) reported that IPo attenuated inflammatory response by reducing serum and tubular tumor necrosis factor-α (TNF-α) expression (12). Renal IPo in the present study was based on the methods described by Liu *et al* (11) and Heusch *et al* (13), employing 10 sec of reperfusion immediately following release of ischemia, followed by 10 sec of ischemia. The process was repeated three times.

Endogenous heat shock proteins (HSPs), categorized into various subfamilies based on their molecular weight, are increasingly expressed upon tissue stress, as a cellular protective mechanism. HSP70 is a chaperone protein which has a key role in stress tolerance (14). HSP27 is a member of the small molecule family of HSPs, and also has an important role in individual stress tolerance at the cellular level and maintenance of integrity (15). Heme oxygenase-1 (HO-1) has been the focus of research in organ transplantation and protection, due to its anti-oxidant and anti-inflammatory activity, as well as the ability to improve microcirculation, inhibit immunological rejection and induce immunological tolerance (16). As an endogenous protective mechanism, the expression of HSP70, HSP27 and HO-1 protect against the progression of IR. Zhang *et al* (17) showed that HSP levels, particularly those of HSP70, HSP27 and HO-1, are highly sensitive to IRI in rat kidneys. Several studies have shown that postconditioning induces expression of HSPs and is protective to the brain and lung (18,19).

The potential application of HSP induction by IPo in the kidney has not been demonstrated, to the best of our knowledge. The present study was designed to determine whether IPo induced higher expression levels of HSP70, HSP27 and HO-1, thereby attenuating renal lipid peroxidation, inflammatory responses and cellular apoptosis, and reducing IRI in the kidneys of rats.

## Materials and methods

### Animals

Male Sprague Dawley rats (n=140), weighing 250–280 g, aged 6–8 weeks, were obtained from the Hebei Laboratory Animal Center (Hebei, China). Rats were housed in a standard environment, under a 12-h light/dark cycle, with access to water and a standard laboratory diet *ad libitum*. All procedures and protocols used in the present study were approved by the Experimental Animal Ethics Committee of Hebei Medical University (Hebei, China), and the guidelines of the National Institutes of Health Guide for the Care and Use of Laboratory Animals were followed.

### Experimental protocol

The rats (n=140) were anesthetized by ether inhalation. Briefly, the peritoneal cavity was opened through a midline incision. Both kidneys were separated and the bilateral renal pedicles were occluded for 45 min using an atraumatic mini-clamp, followed by reperfusion of various durations (1, 3, 6, 12, 24 or 48 h; n=5/time point) (IR group) (11). One group of rats received three cycles of ischemia (10 sec) followed by 10 sec reperfusion following the 45-min ischemia, but prior to restoring full perfusion (IPo group) (11,13). Another group of rats (n=35) was subjected to the IPo procedure, using 100 mg/kg quercetin (HSP inhibitor), injected intraperitoneally at 1 h prior to ischemia (quercetin + IPo group; n=35). Control rats receiving sham operations were used as the negative controls. In these animals, the kidneys were exposed bilaterally for 45 min through a midline incision, but without clamping their pedicles (sham group). Animals in the four groups were sacrificed at the end of ischemia (T0) (corresponding to the end of IPo) and at 1, 3, 6, 12, 24 and 48 h (T1-6) of reperfusion (n=5 rats at each time-point).

### Serum and kidney specimens

Serum was extracted from cardiac blood and kidneys were removed at each time-point. Serum samples were stored at −20°C for biochemical analysis for creatinine (Cr), blood urea nitrogen (BUN) and expression level analysis of TNF-α. Tissue samples were divided into two parts. One part of each specimen was stored at −80°C for measurements of *HSP70*, *HSP27*, *HO-1* and *caspase-3* mRNA by quantitative polymerase chain reaction (qPCR), as well as determination of malondialdehyde (MDA) content and superoxide dismutase (SOD) activity. The other part of each specimen was fixed in 4% paraformaldehyde and embedded in paraffin for histopathology, apoptosis and immunohistochemical analyses.

### RNA isolation, reverse transcription and qPCR

Total RNA was extracted from 50 mg of each renal tissue specimen using TRIzol™ reagent (Invitrogen Life Technologies, Carlsbad, CA, USA) according to the manufacturer’s instructions. RNA purity and content were detected by measuring the optical density (OD) ratio at 260/280 nm using a 756-ultraviolet spectrophotometer (Aucy Technology Instrument Co., Ltd., Shanghai, China). Pure RNA from the reverse transcription was determined by a score of 1.8–2.0 from the 260/280 ratio. Random primers (Promega Corporation, Madison, WI, USA) were used to synthesize first-strand cDNA with Moloney murine leukemia virus Reverse Transcriptase (Promega Corporation). The cDNA was then amplified by qPCR using a Hot Start Fluorescent PCR Core Reagent kit (SYBR^®^ Green I) (Boston Biomedical, Cambridge, MA, USA) and the ABI 7300 Real-Time PCR system (Applied Biosystems Life Technologies, Foster City, CA, USA). Specific primers for HSP70, HO-1, HSP27 and caspase-3 are listed in Table I. qPCR was carried out as follows: Initial denaturation at 96°C for 4 min, followed by 40 cycles of amplification at 94°C for 30 sec, hybridization at 58°C for 30 sec and extension at 72°C for 30 sec. Relative mRNA in each sample was then quantified automatically by reference to the standard curve constructed each time according to SDS v1.3 software (Applied Biosystems Life Technologies). The mRNA levels were calculated with reference to external standard curves constructed by plotting the log number of 10-fold serially diluted cDNA samples against the respective threshold cycle by the second derivative maximum method. The expression of mRNA levels in each sample was normalized against the mRNA expression levels of GAPDH.

### Immunohistochemistry

Immunohistochemical staining was performed using rabbit antibodies against rat HSP70 (BS2741; Bioworld Technology, Inc., St. Louis Park, MN, USA), HO-1 (BS-0827R; Beijing Bioss Biotechnology Co., Ltd. Beijing, China), HSP27 (BS3435; Bioworld Technology, Inc.) or nuclear factor kappa-light-chain-enhancer of activated B cells p65 (NF-κB-p65, BS1253; Bioworld Technology, Inc.) on paraffin sections according to the manufacturer’s instructions. The staining was analyzed using the Leica Q-500 Image Analysis system (Leica Microsystems GmbH, Wetzlar, Germany).

### Terminal deoxynucleotidyl transferase-mediated dUTP nick-end labeling (TUNEL) assay

Apoptosis in kidney cells was identified by TUNEL assays, performed according to the manufacturer’s instructions (Boehringer Ingelheim, Mannheim, Germany). Apoptotic renal tubular epithelial cells were examined by light microscopy (BX53; Olympus Corporation, Tokyo, Japan) at ×400 magnification. The apoptotic index (AI) was defined as the percentage of stained cells/high-power field.

### MDA content and SOD activity

The MDA levels and SOD activity in nephridial tissues were detected using thiobarbituric acid (TBA) and xanthine oxidase methods, according to the manufacturer’s instructions (Nanjing Jiancheng Bioengineering Institute, China). The homogenate (0.1 ml) was used to detect the MDA content. The condensation of MDA and TBA resulted in a red product, with a maximum absorption peak at 532 nm (NanoDrop2000 spectrophotometer; Thermo Fisher Scientific, Wilmington, DE, USA). The MDA content was calculated by measuring the absorbance at 532 nm and expressed as nmol/mg protein (nmol/mg prot). SOD activity was determined by detecting the absorbance at 550 nm. SOD activity was expressed as U/mg prot.

### Detection of TNF-α by enzyme-linked immunosorbent assay (ELISA)

The levels of TNF-α in the serum were measured by ELISA according to the manufacturer’s instructions (Nanjing Jiancheng Bioengineering Institute, Nanjing, China).

### Statistical analysis

Values are expressed as the mean ± standard deviation. Statistical analyses were performed using SPSS 13.0 (SPSS, Inc., Chicago, IL, USA). Analysis of variance was conducted for comparison of parameters among groups and the Student-Newman-Keuls test for comparison of parameters between two groups after normality testing (quantile-quantile plot, Q-Q plot) and tests for homogeneity of variance (Levene’s test). P<0.05 was considered to indicate a statistically significant difference.

## Results

### IPo increases expression of HSPs

**Figure 1:**
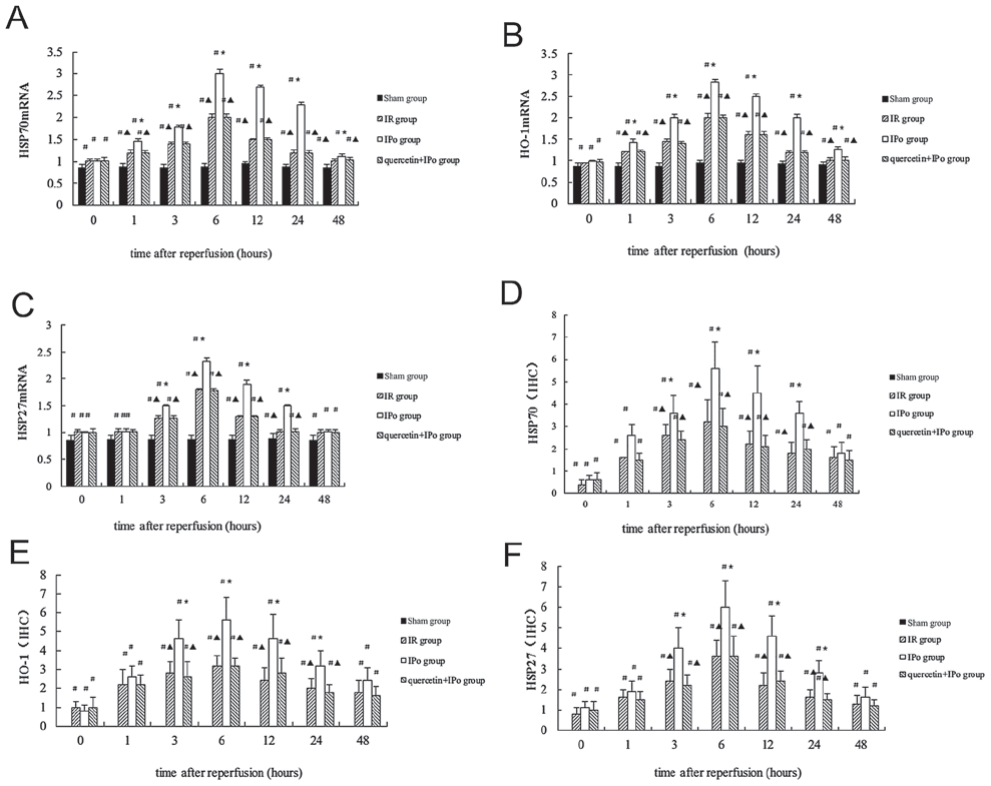
Relative expression of (A) HSP70 mRNA, (B) HO-1 mRNA, (C) HSP27 mRNA, (D) HSP70 protein, (E) HO-1 protein and (F) HSP27 protein, in renal tissue immediately following reperfusion (0 h) or 1, 3, 6, 12, 24 and 48 h after reperfusion (mRNA and protein levels were detected by quantitative polymerase chain reaction and immunohistochemistry). In D-F, the value of the sham group was defined as 0, and the values of the other groups were presented relatively to the sham group. ^#^P<0.05, as compared with the sham group; ^*^P<0.05, as compared with IR group; ^▲^P<0.05, as compared with the IPo group. HSP, heat shock protein; HO-1, heme oxygenase-1; S, control group; IR, ischemia-reperfusion; IPo, ischemic postconditioning; IHC, immunohistochemistry.

Expression levels of HSP70, HSP27 and HO-1 in the kidney were induced by IR at the mRNA and protein level. The expression levels were further elevated by IPo at each time-point, reaching a peak at 6 h after reperfusion. Quercetin inhibited the IPo-mediated increases of HSP70, HSP27 and HO-1 mRNA and protein levels by (Fig. 1A–F).

### Expression of HSPs is involved in the reduction of renal IRI by IPo

**Figure 2:**
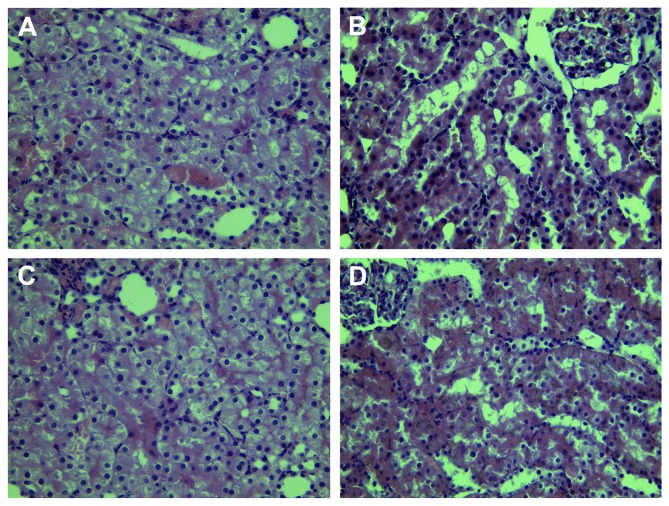
Microphotographs of kidney tissues in the four experimental groups at 6 h post-reperfusion (hematoxylin and eosin stain; magnification, ×400). (A) Control group showed no obvious morphological changes. (B) Ischemia-reperfusion group showed severe pathological and morphological changes, tubular dilatation, cellular edema, with partly visible necrosis and tubular cells. Protein accumulation was detected in the fluid within lumen, together with perivascular dilatation and congestion. (C) The IPo group showed an attenuation of the pathological changes. (D) Quercetin + IPo attenutated the renoprotective effects of IPo. IPo, ischemic postconditioning.

**Figure 3:**
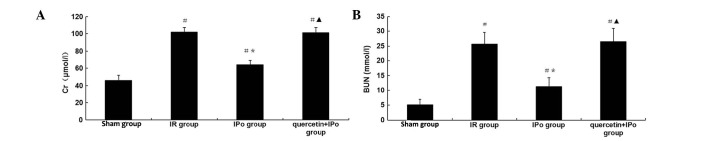
(A) Creatinine and (B) blood urea nitrogen levels in the serum in four groups at 6 h post-reperfusion. ^#^P<0.05, as compared with the sham group; ^*^P<0.05, as compared with the IR group; ^▲^P<0.05, as compared with IPo group. Cr, creatinine; BUN, blood urea nitrogen; S, control group; IR, ischemia-reperfusion; IPo, ischemic postconditioning.

To assess functional renal impairment, changes in renal pathology were observed by microscopy, and levels of Cr and BUN were measured in the serum. Renal IR led to severe pathological and morphological changes, including tubular dilatation and cellular edema, with partly visible necrosis and tubular cells. Protein accumulation in the fluid within lumen, perivascular dilatation and congestion (Fig. 2) as well as increased serum Cr expression levels (102±5 vs. 46±6 μmol/l, P<0.05) and BUN (25.7±3.9 vs. 5.1±1.9 mmol/l, P<0.05) concentrations at 6 h after reperfusion were also observed. IPo attenuated the pathological changes and decreased Cr expression levels (64±5 vs. 102±5 μmol/l, P<0.05) and BUN concentrations (11.3±3.0 vs. 25.7±3.9 mmol/l, P<0.05) (Fig. 3A and B). The renoprotective effects of IPo were significantly attenuated by quercetin (Cr, 101±6 vs. 64±5 μmol/l, P<0.05; BUN, 26.5±4.5 vs. 11.3±3.0, P<0.05).

### Upregulation of HSPs mediates a decrease of MDA content and increase of SOD activity by IPo

**Figure 4:**
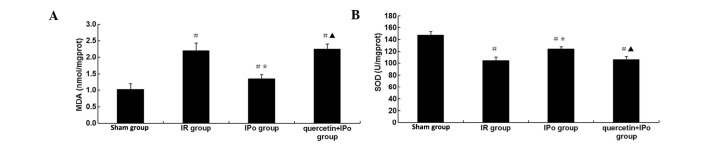
(A) MDA content and (B) SOD activity in the four experimental groups at 6 h post-reperfusion. ^#^P<0.05, as compared with the sham group; ^*^P<0.05, as compared with IR group; ^▲^P<0.05, as compared with the IPo group. S, control group; IR, ischemia-reperfusion; IPo, ischemic postconditiong; MDA, malondialdehyde; SOD, superoxide dismutase.

To assess the levels of lipid peroxidation associated with renal IR, the MDA content and SOD activity in kidney tissue were determined. Following 6 h of reperfusion, the MDA content was significantly increased (2.20±0.23 vs. 1.02±0.19 nmol/mg prot, P<0.05), while the SOD activity was significantly decreased (104±6 vs. 147±6 U/mg prot, P<0.05). IPo attenuated the pathology associated with lipid peroxidation of renal IR (MDA 1.35±0.13 vs. 2.20±0.23 nmol/mg prot, P<0.05; SOD 124±4 vs. 104±6 U/mg prot, P<0.05). This effect was significantly restrained by quercetin (MAD 2.25±0.16 vs. 1.35±0.13 nmol/mg prot, P<0.05; SOD 106±5 vs. 124±4 U/mg prot, P<0.05) (Fig. 4).

### Upregulation of HSPs mediates decrease in NF-κB and TNF-α levels by IPo

**Figure 5:**
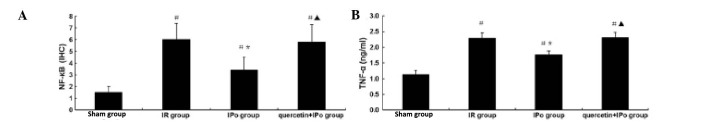
(A) Expression of NF-κB and (B) TNF-α expression levels, in the four experimental groups at 6 h post-reperfusion. ^#^P<0.05, as compared with the sham group; ^*^P<0.05, as compared with the IR group; ^▲^P<0.05, as compared with the IPo group. S, control group; IR, ischemia-reperfusion; IPo, ischemic postconditiong; IHC, immunohistochemistry; TNF-α, tumor necrosis factor-α; NF-κB, renal nuclear factor kappa-light-chain-enhancer of activated B cells.

To determine the extent of pathological inflammation in renal IR, the renal tissue expression levels of NF-κB and the serum expression levels of TNF-α were evaluated. After 6 h of reperfusion, the NF-κB expression (6.0±1.4 vs. 1.5±0.5, P<0.05) in the kidney and the TNF-α levels (2.29±0.18 vs. 1.13±0.14 ng/ml, P<0.05) in the serum were significantly increased. IPo attenuated the inflammation due to renal IR (NF-κB expression, 3.4±1.1 vs. 6.0±1.4, P<0.05; TNF-α levels, 1.76±0.13 vs. 2.29±0.18 ng/ml, P<0.05). The effect by IPo was significantly inhibited by quercetin (NF-κB expression, 5.8±1.8 vs. 3.4±1.1. P<0.05; TNF-α levels, 2.31±0.17 vs. 1.76±0.13 ng/ml, P<0.05) (Fig. 5).

### Upregulation of HSPs reduces apoptosis of renal tubular epithelial cells by IPo

**Figure 6:**
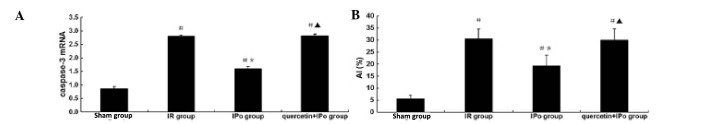
(A) Expression of *caspase-3* mRNA and (B) AI, in the four experimental groups at 6 h post-reperfusion. ^#^P<0.05, as compared with the sham group; ^*^P<0.05, as compared with the IR group; ^▲^P<0.05, as compared with the IPo group. AI, apoptotic index; S, control group; IR, ischemia-reperfusion; IPo, ischemic postconditioning.

**Figure 7:**
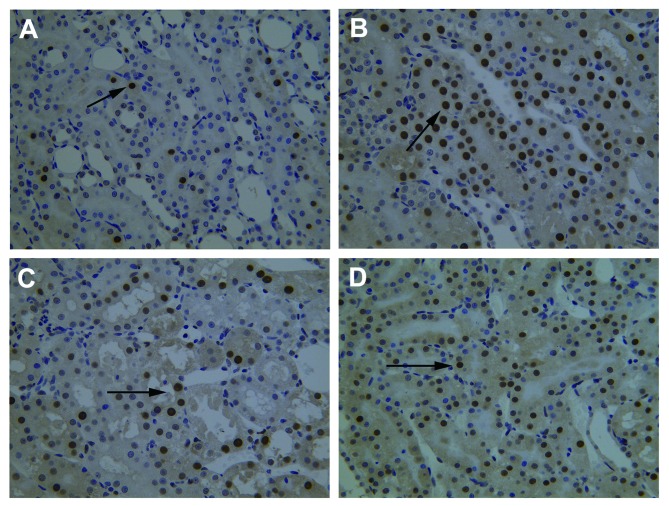
TUNEL staining. Renal apoptosis in the four experimental groups at 6 h post-reperfusion (magnification, ×400). (A) Control group, (B) Ischemic-reperfusion group, (C) Ischemic postconditioning (IPo) group and (D) quercetin + IPo. Arrows indicate TUNEL-positive renal tubular epithelial cells. IPo, ischemic postconditioning; TUNEL, terminal deoxynucleotidyl transferase dUTP nick end labeling.

To observe apoptosis and to evaluate the AI, the expression of *caspase-3* mRNA in renal tubular epithelial cells was assessed by qPCR and TUNEL assays. 6 h after reperfusion, the expression of *caspase-3* mRNA (2.80±0.04 vs. 0.86±0.08, P<0.05) and AI (30.5±4.1 vs. 5.6±1.5%, P<0.05) were significantly increased. An increase of TUNEL-positive renal tubular epithelial cells was observed. IPo decreased the expression of *caspase-3* mRNA (1.60±0.08 vs. 2.80±0.04, P<0.05) and AI (19.3±4.4 vs. 30.5±4.1%, P<0.05), and fewer TUNEL-positive renal tubular epithelial cells were observed. The decrease of apoptosis in the IPo-treated group was significantly attenuated by quercetin (*caspase-3* mRNA, 2.82±0.06 vs. 1.60±0.08, P<0.05; AI 29.9±4.8 vs. 19.3±4.4%, P<0.05) (Fig. 6A and B; Fig. 7).

## Discussion

HSPs have been identified to have various biological functions with protective effects in cells. Previous studies have demonstrated the association of HSPs with the effects of organ IR (17). In the present study, the expression of three HSPs was observed at various time-points following IR. The results suggested that IPo induced higher expression of these proteins.

Quercetin is an inhibitor of HSPs, which acts by interfering with their transcription (20,21). In the present study, quercetin was injected intraperitoneally (100 mg/kg) at 1 h prior to the surgically-induced ischemia, based on methods described previously by Yang *et al* (22) and Yao *et al* (23). Significant increases occurred in the HSP expression in the IPo group as compared with the IR group. However, no differences were detected between the quercetin + IPo group and the IR group. The results indicated that quercetin inhibited IPo-induced HSP expression.

An induction of several HSPs in nephridial tissue following IR was observed, which was seen as a protective mechanism against functional injury to the cells. These results were consistent with those of Zhang *et al* (17), who used gene microarrary analysis to report an increased expression of 21 genes, including HSP70 (43-fold), HSP27 (12-fold) and HO-1 (10-fold), in rat kidneys subjected to early IRI. Furthermore, at each time-point, the expression levels of HSPs were significantly higher in the IPo group as compared with the IR group at the corresponding time-point. The results also indicated that the expression of HSPs was, in part, time-dependent. HSP expression in the tissues in response to stress stimuli peaked at 6 h post-reperfusion, but decreased at 24 h following reperfusion, suggesting activation of the endogenous protective mechanism early during IRI, in order to protect cellular functions.

As HSP expression levels peaked at 6 h post-reperfusion, the serum Cr and BUN levels, renal tubular epithelial cell apoptosis and histopathological changes were determined to confirm the protective effects of IPo. The excessive generation of oxygen radicals causing lipid peroxidation of cell membranes, protein and enzyme oxidation and irreversible DNA changes, lead to the inactivation of key cellular functions and ultimately to cell death (2). HSP70 regulates the activities of anti-oxidative enzymes by protective SOD activity (24), attenuating lipid peroxidation (25) and repairing proximal tubule structure following renal ischemia (26). The excessive formation of oxygen radicals is known to destroy the equilibrium of oxidation-reduction reactions in an organism. A previous study (27) has suggested that HSP70 regulates the cellular redox status by modulating glutathione-associated enzyme activities.

HO-1 has been the focus of research in organ transplantation and protection, as it has anti-oxidant and anti-inflammatory functions, as well as the ability to improve microcirculation, inhibit immunological rejection and induce immunological tolerance. Overexpression of HO-1 has been associated with a decreased generation of oxygen radicals, increased SOD levels in serum, attenuated oxidative stress, decreased infiltration of neutrophilic granulocytes and release of inflammatory factors, as well as protection against IRI in the kidney (28,29) and other organs (30–32).

HSP27 overexpression in tissues has been shown to inhibit the release of proinflammatory factors, such as TNF-α and macrophage inflammatory protein 2 (MIP2), as well as the infiltration of neutrophilic granulocytes; these events are known to protect against IRI-induced damage (33 35). However, a previous study suggested that a systemic increase of HSP27, instead of a local increase, in transgenic mice counteracts this protection by exacerbating renal and systemic inflammation (36). HSP27 has been shown to inhibit the disassociation of actin and microfibrils, offering protection and stabilization to the cytoskeleton (34,35). This function of HSP27 is important in the tolerance of individual cells and organs to different stresses by maintaining the integrity of the endothelium and epithelium.

The MDA content reflects the degree of lipid oxidative reactions, whereas the SOD activity may reflect the ability of the body to scavenge oxygen free radicals. In the present study, MDA levels were decreased and the activity of SOD was increased following renal IPo, which was reversed in the presence of quercetin. This suggested that IPo elevated the expression of HSPs and attenuated lipid peroxidation in renal IRI.

Previous studies have shown that HSP70 and HO-1 can inhibit the activation of NF-κB, increase the expression of nuclear factor of kappa light polypeptide gene enhancer in B-cells inhibitor, downregulate the expression of TNF-α and attenuate IRI (37,38). In the present study, IPo induced the expression of HSPs and reduced the levels of NF-κB and TNF-α. In the presence of quercetin these effects of IPo were inhibited. These findings suggested that IPo attenuated inflammatory reactions, following renal IR, and that the renal protection was associated with the expression of HSPs.

In previous studies, HSP70, HSP 27 and HO-1 (34,39,40) were identified to reduce organ IRI through the inhibition of mitochondrial cytochrome C release, caspase-3 activation, inhibition of B-cell lymphoma 2 (Bcl-2)-associated X, elevation of Bcl-2 and Bcl-2 extra large gene expression and reduction of apoptosis. In the present study, analysis of renal tubular epithelial cell apoptosis indicated that in the IPo group, the *caspase-3* mRNA levels and the AI decreased. The addition of quercetin attenuated these effects, followed by a decrease in the expression of HSPs. This suggested that IPo increased the expression of HSPs, reduced apoptosis, thereby reducing renal IRI.

In conclusion, the present study indicated that IPo induced HSP70, HSP27 and HO-1 expression. The subsequent reduction of the generation of superoxide anions and peroxides upon sudden reperfusion following ischemia, attenuating lipid oxidation, reducing the levels of NF-κB and TNF-α, inflammatory response, and cellular apoptosis, as well as renal IRI.

## Figures and Tables

**Table I:** Primer sequences used in quantitative polymerase chain reaction.

Primer		Sequence 5′ to 3′
HSP70	Forward	GGGTTTGGGTACTTTGGTTA
	Reverse	CCCATAAGTTGGGAAACAGT
HO-1	Forward	GAGGAGATAGAGCGAAACAAGC
	Reverse	GTGGCTGGT GTGTAAGGGAT
HSP27	Forward	AGCAGCGGTGTG TCAGAGAT
	Reverse	GCCTTCCTTGGTCTTCACTGT
Caspase-3	Forward	GACAACAACGAAACCTCCG
	Reverse	AGGGTTAGCTGCATCGACA
GAPDH	Forward	TGAACGGGAAGCTCACTGG
	Reverse	GCTTCACCACCTTCTTGATGTC

HSP, heat shock protein; HO, heme oxygenase.

## References

[1] Serviddio G, Romano AD, Gesualdo L (2008). Postconditioning is an effective strategy to reduce renal ischaemia/reperfusion injury. Nephrol Dial Transplant.

[2] Kunduzova OR, Bianchi P, Pizzinat N (2002). Regulation of JNK/ERK activation, cell apoptosis, and tissue regeneration by monoamine oxidases after renal ischemia-reperfusion. FASEB J.

[3] Akoh JA (2011). Transplant nephrectomy. World J Transplant.

[4] Zhao ZQ, Corvera JS, Halkos ME (2003). Inhibition of myocardial injury by ischemic postconditioning during reperfusion: comparison with ischemic preconditioning. Am J Physiol Heart Circ Physiol.

[5] Penna C, Tullio F, Moro F (2010). Effects of a protocol of ischemic postconditioning and/or captopril in hearts of normotensive and hypertensive rats. Basic Res Cardiol.

[6] Wever KE, Menting T, Masereeuw R (2012). Local and remote ischemic postconditionings have synergistic protective effects on renal ischemia-reperfusion injury. Transplantation.

[7] Wang JY, Shen J, Gao Q (2008). Ischemic postconditioning protects against global cerebral ischemia/reperfusion-induced injury in rats. Stroke.

[8] Liu KX, Li YS, Huang WQ (2009). Immediate postconditioning during reperfusion attenuates intestinal injury. Intensive Care Med.

[9] Lønborg J, Kelbaek H, Vejlstrup N (2010). Cardioprotective effects of ischemic postconditioning in patients treated with primary percutaneous coronary intervention, evaluated by magnetic resonance. Circ Cardiovasc Interv.

[10] Deftereos S, Giannopoulos G, Tzalamouras V (2013). Renoprotective effect of remote ischemic post-conditioning by intermittent balloon inflations in patients undergoing percutaneous coronary intervention. J Am Coll Cardio.

[11] Liu JJ, Zhao YL, Zhang YD (2007). Effects of ischemic postconditioning on the renal ischemia-reperfusion injury in rats. Chin J Anesthesiol.

[12] Miklós Z, Kürthy M, Degrell P (2012). Ischaemic postconditioning reduces serum and tubular TNF-α expression in ischaemic-reperfused kidney in healthy rats. Clin Hemorheol Microcirc.

[13] Heusch G, Büchert A, Feldhaus S, Schulz R (2006). No loss of cardioprotection by postconditioning in connexin 43-deficient mice. Basic Res Cardiol.

[14] Stricher F, Macri C, Ruff M, Muller S (2013). HSPA8/HSC70 chaperone protein: Structure, function, and chemical targeting. Autophagy.

[15] Ziemann E, Zembroñ-Lacny A, Kasperska A (2013). Exercise training-induced changes in inflammatory mediators and heat shock proteins in young tennis players. J Sports Sci Med.

[16] Morse D, Choi AM (2002). Heme oxygenase-1: the ‘emerging molecule’ has arrived. Am J Respir Cell Mol Biol.

[17] Zhang PL, Lun M, Schworer CM (2008). Heat shock protein expression is highly sensitive to ischemia-reperfusion injury in rat kidneys. Ann Clin Lab Sci.

[18] Xing B, Chen H, Zhang M (2008). Ischemic postconditioning inhibits apoptosis after focal cerebral ischemia/reperfusion injury in the rat. Stroke.

[19] Xu B, Gao X, Xu J (2011). Ischemic postconditioning attenuates lung reperfusion injury and reduces systemic proinflammatory cytokine release via heme oxygenase 1. J Surg Res.

[20] Manwell LA, Heikkila JJ (2007). Examination of KNK437- and quercetin-mediated inhibition of heat shock-induced heat shock protein gene expression in Xenopus laevis cultured cells. Comp Biochem Physiol A Mol Integr Physiol.

[21] Khomenko IP, Bakhtina LY, Zelenina OM (2007). Role of heat shock proteins HSP70 and HSP32 in the protective effect of adaptation of cultured HT22 hippocampal cells to oxidative stress. Bull Exp Biol Med.

[22] Yang CW, Li C, Jung JY (2003). Preconditioning with erythropoietin protects against subsequent ischemia-reperfusion injury in rat kidney. FASEB J.

[23] Yao K, Rao H, Wu R, Tang X, Xu W (2006). Expression of Hsp70 and Hsp27 in lens epithelial cells in contused eye of rat modulated by thermotolerance or quercetin. Mol Vis.

[24] Lepore DA, Knight KR, Anderson RL, Morrison WA (2001). Role of priming stresses and Hsp70 in protection from ischemia-reperfusion injury in cardiac and skeletal muscle. Cell Stress Chaperones.

[25] Siu PM, Wang Y, Alway SE (2009). Apoptotic signaling induced by H2O2-mediated oxidative stress in differentiated C2C12 myotubes. Life Sci.

[26] Bidmon B, Endemann M, Müller T (2000). Heat shock protein-70 repairs proximal tubule structure after renal ischemia. Kidney Int.

[27] Guo S, Wharton W, Moseley P, Shi H (2007). Heat shock protein 70 regulates cellular redox status by modulating glutathione-related enzyme activities. Cell Stress Chaperones.

[28] Katavetin P, Inagi R, Miyata T (2007). Erythropoietin induces heme oxygenase-1 expression and attenuates oxidative stress. Biochem Biophys Res Commun.

[29] Ferenbach DA, Ramdas V, Spencer N (2010). Macrophages expressing heme oxygenase-1 improve renal function in ischemia/reperfusion injury. Mol Ther.

[30] Park J, Kang JW, Lee SM (2013). Activation of the cholinergic anti-inflammatory pathway by nicotine attenuates hepatic ischemia/reperfusion injury via heme oxygenase-1 induction. Eur J Pharmacol.

[31] Saeki I, Matsuura T, Hayashida M, Taguchi T (2011). Ischemic preconditioning and remote ischemic preconditioning have protective effect against cold ischemia-reperfusion injury of rat small intestine. Pediatr Surg Int.

[32] Jia XM, Zhou ZX, Huang JJ, Chu W, Guan QH (2007). Protective effects of the induction of heme oxygenase-1 on ischemia reperfusion lung injury: *in vivo* experiment with rats. Zhonghua Yi Xue Za Zhi.

[33] Park SW, Chen SW, Kim M, D’Agati VD, Lee HT (2009). Human heat shock protein 27-overexpressing mice are protected against acute kidney injury after hepatic ischemia and reperfusion. Am J Physiol Renal Physiol.

[34] Chen SW, Park SW, Kim M (2009). Human heat shock protein 27 overexpressing mice are protected against hepatic ischemia and reperfusion injury. Transplantation.

[35] Park SW, Chen SW, Kim M, D’Agati VD, Lee HT (2010). Selective intrarenal human A1 adenosine receptor overexpression reduces acute liver and kidney injury after hepatic ischemia reperfusion in mice. Lab Invest.

[36] Chen SW, Kim M, Kim M (2009). Mice that overexpress human heat shock protein 27 have increased renal injury following ischemia reperfusion. Kidney Int.

[37] Kuboki S, Schuster R, Blanchard J (2007). Role of heat shock protein 70 in hepatic ischemia-reperfusion injury in mice. Am J Physiol Gastrointest Liver Physiol.

[38] Devey L, Mohr E, Bellamy C (2009). c-Jun terminal kinase-2 gene deleted mice overexpress hemeoxygenase-1 and are protected from hepatic ischemia reperfusion injury. Transplantation.

[39] Ke B, Shen XD, Gao F (2009). Small interfering RNA targeting heme oxygenase-1 (HO-1) reinforces liver apoptosis induced by ischemia-reperfusion injury in mice: HO-1 is necessary for cytoprotection. Hum Gene Ther.

[40] Manucha W, Vallés PG (2008). Cytoprotective role of nitric oxide associated with Hsp70 expression in neonatal obstructive nephropathy. Nitric Oxide.

